# Current Status and Future Challenges of Liquid Biopsy

**DOI:** 10.3390/cells14242000

**Published:** 2025-12-16

**Authors:** Shuta Ohara, Kenichi Suda

**Affiliations:** 1 Division of Thoracic Surgery, Department of Surgery, Kindai University Faculty of Medicine, 1-14-1 Mihara-dai, Minami-ku, Sakai 590-0197, Japan; 154148@med.kindai.ac.jp; 2 Department of General Thoracic Surgery, Kindai University Nara Hospital, Otoda-cho 1248-1, Ikoma 630-0293, Japan; 3 Division of Thoracic Surgery, Izumi City General Hospital, 4-5-1 Wake-cho, Izumi 594-0073, Japan

Liquid biopsy has rapidly advanced as an innovative tool in precision oncology, allowing clinicians to capture the dynamic molecular and/or quantitative changes in tumors with minimal invasiveness. By analyzing various tumor-derived or tumor-affected components—such as circulating tumor DNA (ctDNA), cell-free DNA (cfDNA), cell-free RNA (cfRNA), circulating microRNA (cmiRNA), circulating tumor cells (CTCs), tumor-educated platelets (TEPs), and extracellular vesicles—liquid biopsies enable detection of minimal residual disease (MRD) (associated with recurrence risk after curative-intent treatment), tumor burden monitoring, earlier detection of disease recurrence/disease progression, and therapeutic guidance by detecting driver mutation, resistant mutation, and tumor mutation burden.

Six articles have been published in this Special Issue of *Cells* entitled “Current Status and Future Challenges of Liquid Biopsy”. These papers highlighted technological advances of liquid biopsies as well as enhanced translational and clinical implications of liquid biopsies in cancer care. It is of note that these articles covered a wide range of solid tumors, including breast, lung, colorectal, and biliary tract cancers, indicating the wide applicability of this new technology throughout the field of oncology.

Among six papers, three were review/perspective articles focusing on the roles of ctDNA as a tool for MRD detection in early-stage disease. Vlataki et al. reviewed the potential clinical utility and future perspectives of ctDNA in early-stage breast cancer. They focused on the roles of ctDNA in early detection/diagnosis of breast cancer, as prognostic/predictive biomarkers for patients who receive neoadjuvant treatment, and as tools to detect MRD and perform disease surveillance after surgery [1]. Focusing on lung cancer, especially for patients receiving neoadjuvant chemo-immunotherapy (plus adjuvant immunotherapy), we also summarized the potential roles of ctDNA analysis in this Special Issue [2]. Reported studies have emphasized the utility of ctDNA clearance after neoadjuvant treatment as a predictor of pathological response, and that landmark ctDNA (MRD status) predicts the risk of disease recurrence, and longitudinal ctDNA analysis would be useful to detect disease recurrence prior to radiological recurrence. Lastly, Abidoye et al. provided a comprehensive review of ctDNA-based MRD detection in colorectal cancer patients [3]. The DYNAMIC trial [4] demonstrated the feasibility of treatment de-escalation, whereas the GALAXY [5] study confirmed the benefit of adjuvant chemotherapy in MRD-positive patients.

In addition to these review works, Kujala et al. reported the results of their analysis to identify circulating miRNAs that will predict disease recurrence in triple-negative breast cancer (TNBC) [6]. They identified three circulating miRNAs—miR-21-5p, miR-16-5p, and miR-26b-5p—as significant predictors of disease recurrence. They also demonstrated a correlation between serum and tumor miRNAs expression, supporting the use of circulating miRNA signatures as non-invasive biomarkers for recurrence risk stratification in TNBC. Based on their findings, we consider that combining ctDNA with other liquid biopsy modalities, including circulating miRNAs, may improve the sensitivity of MRD detection.

Insufficient sensitivity is a major issue in applying ctDNA-based MRD detection in clinical practice in many solid tumors. CRC is one of the tumor types in which ctDNA readily sheds into the bloodstream, and thus several studies have reported the utility of ctDNA-based MRD analysis in patients with CRC. However, even in patients with CRC, the sensitivity of ctDNA-based MRD detection remains relatively low at ~70% at the landmark time point. To improve the sensitivity of ctDNA detection from the point of technological advance, Sathyanarayana et al. evaluated the impacts of pre-analytical factors, including the utility of the magnetic bead-based cfDNA extraction system [7]. They reported that pre-analytical factors—such as blood collection method, storage temperature, and plasma processing—have a major impact on cfDNA quality. Properly controlling these factors helps minimize genomic DNA contamination and maintain the integrity of cfDNA.

It is of note that the “liquid” does not always indicate plasma. Okuno et al. reported the results of their molecular analysis utilizing cell block specimens obtained from overnight-stored bile in patients with cholangiocarcinoma [8]. Their feasibility study demonstrated a nearly complete concordance between bile-derived and surgically resected specimens, suggesting that bile sampling could serve as a practical and accessible method for molecular profiling when surgical tissue is limited.

Through the studies included in this Special Issue, we found expanding roles and limitation of liquid biopsies from various aspects to further improve patients’ outcomes across diverse tumor types. Currently, ctDNA-based mutation detection, including liquid-based comprehensive genome profiling tests, is widely used in clinical practice. We expect that the next promising phase of liquid biopsy will be its application in daily clinical practice as a tool to detect MRD after overcoming several issues, including its sensitivity. Therefore, further studies are essential in this field, and we thus decided to launch a second edition of this Special Issue (https://www.mdpi.com/journal/cells/special_issues/5F4V5SSIPQ) (accessed on 9 December 2025). We expect that liquid biopsy-based MRD detection and additional approaches to liquid biopsy will be useful components of precision oncology clinical practice in the near future.

## Data Availability

No new data were created or analyzed in this study. Data sharing is not applicable to this article.
